# Comparison of Shear Strength of Metal and Ceramic Orthodontic Brackets Cemented to Zirconia Depending on Surface Treatment: An *In Vitro* Study

**DOI:** 10.1055/s-0039-1694304

**Published:** 2019-08-26

**Authors:** Sibel Cetik, Thaï Hoang Ha, Léa Sitri, Hadrien Duterme, Viet Pham, Ramin Atash

**Affiliations:** 1 Department of Stomatology and Dentistry, Erasmus Hospital and Laboratory of Physiology and Pharmaceutics, Faculty of Medicine, Université Libre de Bruxelles, Brussels, Belgium; 2 Department of Stomatology and Dentistry, Erasmus Hospital, Université Libre de Bruxelles, Brussels, Belgium; 3 Dental Materials Research Center, School of Dentistry, Isfahan University of Medical Sciences, Isfahan, Iran and Department of Stomatology and Dentistry, Erasmus Hospital, Université Libre de Bruxelles, Brussels, Belgium

**Keywords:** zirconia, brackets, cementation, surface treatment

## Abstract

**Objectives** Due to the high demand for all-ceramic restorations, monolithic zirconia restorations are nowadays frequently used. With the demand for adult orthodontic treatments, orthodontists need to be mindful of the quality of their brackets bonding to this type of material, as it requires special conditioning. This study aimed to compare different surface treatments of zirconia when bonding metal or ceramic orthodontic brackets. The objectives are to compare the shear bond strength; the amount of adhesive remaining on the surface of the material; the incidence of adhesive, cohesive, and mixed failures; and the occurrence of zirconia fractures.

**Materials and Methods** Forty monolithic blocks of zirconia of a diameter of 10 mm and a length of 10 mm were prepared and randomly divided into two groups ( *n* = 20): metallic or ceramic brackets. Each group was subsequently divided into two subgroups ( *n* = 10) depending on the surface preparation (laser treatment or airborne particle abrasion): SMB (airborne particle abrasion, metal bracket), SCB (airborne particle abrasion, ceramic bracket), LMB (laser; metal bracket), and LCB (laser, ceramic bracket). The samples were tested for shear bond strength using a universal testing machine. The adhesive remnant index and the occurrence of zirconia fractures and different types of failures were assessed by optical and electron microscopy.

**Statistical Analysis** Results were analyzed using analysis of variance.

**Results** The differences were significant between the metallic (SMB, LMB) and ceramic (SCB, LCB) bracket groups with regard to shear bond strength, with respectively 23.29 ± 5.34 MPa, 21.59 ± 4.03 MPa, 20.06 ± 4.05 MPa, and 17.55 ± 3.88 MPa. In terms of surface treatment, no statistical differences were found between the different groups.

**Conclusion** Metal brackets have a greater bond strength than ceramic brackets when cemented to zirconia. The surface treatment of zirconia surface has no influence on the shear bond strength.

## Introduction

The aesthetic demands of patients are increasing, and all-ceramic fixed partial dentures (FPDs) meet their needs. Research has led to the development of zirconium oxide, or zirconia (ZrO _2_ ), a material that presents many advantages such as enhanced aesthetic compared with traditional metal-ceramic restorations, good chemical properties, dimensional stability, and high mechanical strength. Moreover, it presents a Young's modulus of 210 GPa, which is similar to that of stainless steel alloy. 1 2 Its high physical properties come from a phenomenon called “transformation toughening.” 1 2 According to Sailer et al, 3 if an all-ceramic FPD has to be placed in the posterior region, the use of zirconia is recommended. However, when zirconia is used as a framework, these restorations present a higher rate of veneering ceramic chipping compared with metal-ceramic one. The reasons for these chippings are numerous, such as differences in coefficient of thermal expansion between framework and porcelain, firing shrinkage of porcelain, porosities, poor wetting of veneering, flaws on veneering, inadequate framework design to support veneer porcelain, overloading, and fatigue. 4 One alternative to avoid these chipping is to use nonveneering or monolithic zirconia restorations. 4 5

Orthodontists are regularly faced with patients who present monolithic zirconia restorations. But despite all its qualities, 6 7 bonding on zirconia represents a real challenge, 8 9 and as a result the bond failure rate is higher than that with enamel. 10 Practitioners seek to obtain a bond strong enough to reduce bracket detachment from zirconia surfaces. Zirconia has no glass phase, 11 12 so surface preparation using hydrofluoric acid cannot be used to improve bond strength. 4 13 Besides, using this acid in the oral cavity can be dangerous for dental and soft tissues. 8

Laser treatments are constantly evolving, and are used in general practice, for certain treatments in periodontics, as well as in dental surgery, surgery, and other fields. 14 If certain parameters are observed, laser treatment can be used to roughen the surface of a zirconia restoration. For this reason, it has been recommended in some studies. 9 15 Methods such as airborne particle abrasion, 7 9 11 13 16 17 18 19 laser treatment, 7 9 10 11 15 16 17 or even silanization 17 18 have been investigated in previous studies.

Some studies have shown that ceramic brackets are recommended on surfaces like zirconia ahead of metal brackets, 17 yet a recent study has claimed the opposite, finding that metal brackets seemed to adhere more strongly to zirconia surfaces because of their better base surface design and their method of retention. 19

The aim and objective of this study are to evaluate the influence of orthodontic bracket material (metallic or ceramic) and zirconia surface treatment (airborne particle abrasion or laser-etching) on the shear bond strength of these brackets to surface treated monolithic zirconia blocks. We also investigated the amount of residual cement on the blocks after failure by means of the adhesive remnant index (ARI) using an electron microscope and an optical microscope. We then observed the occurrence of adhesive or cohesive failures to determine whether the bonds created between the interfaces were stronger than the bonds within the materials themselves (or vice versa), as well as the occurrence of restoration material fractures.

## Materials and Methods

### Samples Preparation

Forty blocks of polychromatic, super translucent monolithic zirconia (Ceramill Zolid FX Multilayer; Amann Girrbach, Koblach, Austria), shade B2-B3, 10 mm in diameter and 10 mm in length, were prepared, and randomly divided into two groups ( *n* = 20): metallic (Victory Series Low Profile Bracket, Univ L Anterior, 0.022, 3M) and ceramic (Clarity Advanced Ceramic Brackets, Lower Anterior, Roth Rx, 0.022, 3M) ( Table 1 ), and subsequently divided in subgroups: SMB (airborne particle abrasion/metallic brackets), SCB (airborne particle abrasion/ceramic brackets), LMB (laser/metallic brackets), and LCB (laser/ceramic brackets).

**Table 1:** Group names depending on zirconia preparation and type of bracket cemented to the surface of the block

Name of the group	Surface treatment	Bracket type
SMB	Airborne particle abrasion	Metal
SCB	Airborne particle abrasion	Ceramic
LMB	Laser	Metal
LCB	Laser	Ceramic

### Surface Treatment

For half of the samples ( *n* = 20), the surfaces were prepared by airborne particle abrasion using 25 μm aluminum oxide (Basic Master sandblasting unit; Renfert, Hilzingen, Germany) for 20 seconds at 2.5 bar and a distance of 10 mm. 7

For the other half ( *n* = 20), the surfaces were covered with graphite powder (HB pencil) to increase its energy absorption and then subjected to erbium-doped yttrium aluminum garnet (Er:YAG) laser radiation (Fidelis Plus III; Fotona, Ljubljana, Slovenia). The laser was set at a wavelength of 2940 nm, pulse duration of 50μs (SSP), power of 2 W, pulse repetition rate of 10 Hz, and an energy density of 200 mJ. An R14 handpiece was used and equipped with a sapphire tip of a diameter of 0.8 mm. The air/water spray ratio was set at 4/4. The sapphire tip was held perpendicular to the surface of the block at an approximate distance of 0.5 mm. The surface of the zirconia block (78.54 mm ^2^ ) was then subjected to radiation at a speed of around 2 mm/s for 10 seconds using horizontal scanning. 15 All samples were rinsed using the air/water spray for 30 seconds. 12

### Bonding

The bonding steps were performed in accordance with the manufacturer's instructions.

A layer of silane (Clearfil Ceramic Primer Plus, Kuraray Noritake Dental Inc., Osaka, Japan) was applied to the adherend surface of the zirconia block for 20 seconds using an application brush, after which the entire adherend was suitably dried using a moderate, oil-free air spray.

The primer (BrackFix Primer, Voco) was measured into a mixing palette and applied to the surface of the conditioned zirconia surface in a thin, uniform film using a microbrush. Since the primer is photopolymerizable, intense exposure to ambient light was avoided and the intensity of the surgical lighting was reduced during application.

A sufficient quantity of bonding agent (BrackFix; Voco GmbH, Cuxhaven, Germany) was applied to the base surface of each bracket. As soon as the bonding agent had been applied, the bracket was lightly placed on the surface of the zirconia block, its position adjusted, and it was then firmly pressed down. Excess adhesive around the bracket base was delicately removed with a probe without moving the bracket. Lastly, we performed photopolymerization using a curing light (Elipar DeepCure-S, 3M, St Paul, MN, United States) with an intensity of 1470mW/cm ^2^ (–10%/+20%): light oriented either on the interproximal surfaces of the metallic brackets for 10s or perpendicularly to the ceramic brackets for 20 s.

The blocks were then placed in a cold-curing resin (Selacryl Cold powder pink + Selacryl Cold liquid, Selexion) except for the surface where the bracket had been cemented.

### Shear Strength Test

**Fig. 1:**
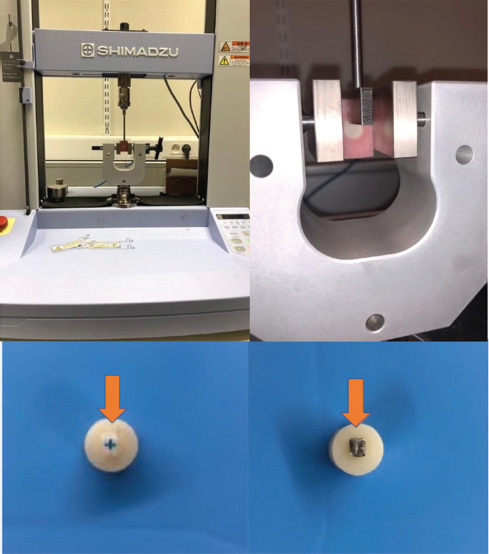
Universal testing machine (Autograph AGS-X, Shimadzu, 1000 N unit) and direction of stress applied to the brackets.

The shear strength test was conducted using a universal testing machine (Autograph AGS-X; Shimadzu, Osaka, Japan). Shear stress was applied in a downwards direction parallel to the surface of the zirconia block at a speed of 0.5 mm/m ( Fig. 1 ).

The load applied was recorded in N and shear strength in MPa. An optical microscope (magnification x20) (Leica; Wetzlar, Germany) was used to determine the ARI score after failure.

### ARI Score

The ARI score is represented by a scale with four levels, from 0 to 3:

0. No adhesive left on the surface
1. Less than half of the adhesive left on the surface
2. More than half of the adhesive left on the surface
3. All of the adhesive left on the surface after failure.

### Type of Failure/Fracture

Using the optical (Leica) and electron (iT 300; Jeol, Akishima, Tokyo, Japan) microscopes, we were able to check for adhesive, cohesive, and mixed failures as well as fractures of the zirconia restoration material. The steps in the protocol were all performed by the same operator.

### Statistical Analysis

Statistical analysis was conducted using IBM SPSS Statistics, version 23 (Statistical Package for Social Sciences; SPSS Inc., Chicago, IL, United States).

## Results

**Fig. 2:**
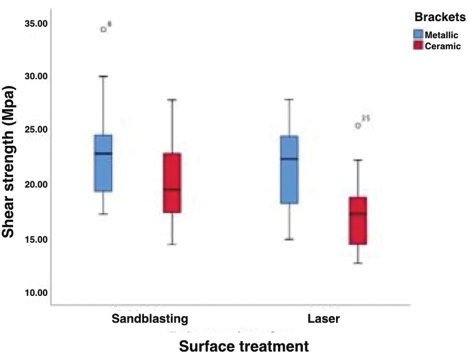
Graph comparing the shear strength (MPa) of the samples depending on surface treatment and type of bracket.

The values obtained on bracket debonding during shear testing are described in Table 2 ( Fig. 2 ).

**Table 2:** Shear strength values (MPa) for the samples of the different groups

Name of the group	SMB	SCB	LMB	LCB
No. 1	24.53	19.29	24.43	14.32
No. 2	19.62	22.83	27.86	16.69
No. 3	19.27	15.13	21.34	18.73
No. 4	19.27	19.46	20.32	12.53
No. 5	17.14	17.29	24.31	25.43
No. 6	22.48	14.29	17.15	17.63
No. 7	23.34	27.89	24.51	14.31
No. 8	30.03	19.45	18.15	22.19
No. 9	19.00	23.21	23.25	15.70
No. 10	23.12	21.83	14.78	18.02

### Shear Strength

Descriptive statistics, including the mean, standard deviation, median, and minimum and maximum values for shear strength (MPa), were calculated. Because this test was not significant ( *p* > 0.05), we accepted the null hypothesis that the variances were equal. The variances were therefore deemed to be similar.

Two-way analysis of variance (ANOVA) was conducted to determine whether there existed a statistically significant difference between the groups. The differences were significant between the metal (SMB, LMB) and ceramic (SCB, LCB) bracket groups with regard to shear bond strength, with respectively 23.29 ± 5.34 MPa, 21.59 ± 4.03 MPa, 20.06 ± 4.05 MPa, and 17.55 3.88 MPa. The SMB group (airborne particle abrasion and metal bracket) showed the highest shear bond strength values, whereas the lowest was that of the LCB group (Er:YAG laser and ceramic bracket).

The ANOVA was significant ( *p* < 0.05) between the different types of bracket. Conversely, the groups treated with airborne particle abrasion or laser-etching did not display any statistically significant differences in terms of their shear strength.

### ARI Score

**Fig. 3:**
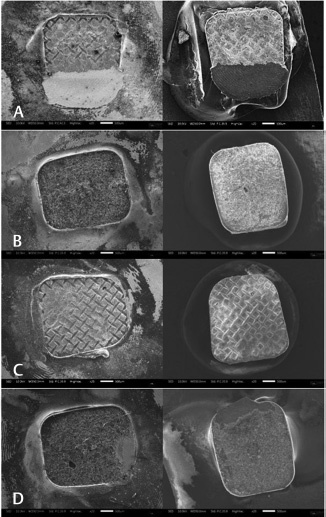
Bracket base of each group (SMB, SCB, LMB, and LCB) and corresponding surface after debonding on electron microscopy (x20). ( **A** ) Sample from SMB group. ( **B** ) Sample from SCB group. ( **C** ) Sample from LMB group. ( **D** ) Sample from LCB group.

The frequency distribution and percentage of ARI scores of the four groups are shown in Table 3 . A sample of blocks and brackets analyzed and used to evaluate the ARI score is shown in Figs. 3 4 .

**Table 3:** Frequency distribution and percentage of adhesive remnant index (ARI) scores of the four groups (n = 10 for each group)

Name of the group	0	1	2	3
SMB	0	0	6 (60%)	4 (40%)
SCB	0	0	3 (30%)	7 (70%)
LMB	0	0	8 (80%)	2 (20%)
LCB	0	1 (10%)	3 (30%)	6 (60%)

**Fig. 4:**
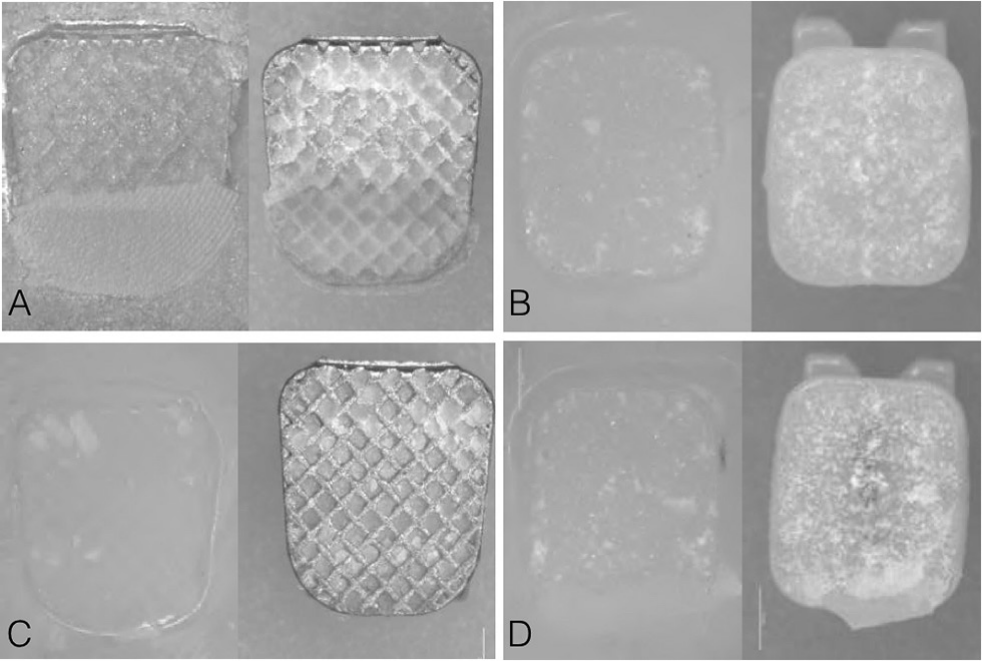
Bracket base of each group (SMB, SCB, LMB, and LCB) and corresponding surface after debonding on optical microscopy (x20). ( **A** ) Sample from SMB group. ( **B** ) Sample from SCB group. ( **C** ) sample from LMB group. ( **D** ) Sample from LCB group.

Statistical analysis in the form of the Kruskal–Wallis test was conducted on the variable “ARI score” and was not significant ( *p* > 0.05; data not shown). Thus, there was no statistically significant difference between the groups.

The LMB group displayed the lowest mean ARI score, followed by the SMB, LCB, and SCB groups. The groups with ceramic brackets had higher mean ARI scores than those with metal brackets. However, after analysis, there was no statistically significant difference between these groups.

### Types of Failures and Fractures

Examination of the samples after bracket debonding revealed mixed fractures and adhesive fractures between the bracket and the resin. No cohesive fracture within the zirconia or the brackets was observed.

## Discussion

The objective of this study is to evaluate the shear bond strength of metal and ceramic orthodontic brackets bonded to monolithic zirconia blocks, with their surface treated with two different treatments (Er:YAG laser treatment, and airborne particle abrasion using 25μm aluminum oxide). The aims are to evaluate the influence of brackets type and influence of zirconia surface treatment on the shear bond strength of orthodontic brackets to zirconia surfaces.

It is difficult to achieve a long-term bond to ceramic surfaces. The findings of previous studies confirm that applying hydrofluoric acid to zirconia does not result in effective retention. 4 13 Other method of surface treatment had to be applied. To date, several studies have demonstrated the benefit of airborne particle abrasion using aluminum oxide (Al _2_ O _3_ ), 7 11 13 16 17 20 since abrasion with particles of aluminum oxide does indeed roughen the surface of restorations. However, we cannot compare the results of this study with those of previously studies, since the grain size chosen are different.

Laser is employed in multiple fields of dentistry, 14 including periodontics, dental surgery, and minor surgery. Several studies have used laser treatment to prepare a surface for the bonding of a bracket. For zirconia restorations, it has been demonstrated that Er:YAG laser treatment is recommended ahead of others such as Nd:YAG and CO _2_ , which create micro-cracks. 9 10 If the parameters discussed in the material and method section are adhered to, Er:YAG can be used to roughen the surface of zirconia without altering its structure.

We did not observe any statistically significant difference in this study between the samples based on the surface treatment they were subjected to, although the highest mean shear strength was achieved by the airborne particle abrasion group.

Some studies have shown that ceramic brackets are recommended for surfaces like zirconia ahead of metal brackets, 17 yet a recent study has claimed the opposite, finding that metal brackets seemed to adhere more strongly to zirconia surfaces because of their better base surface design and their method of retention. 19

In this study, the samples that were given metal brackets displayed greater shear strength, and a statistically significant difference was indeed found between the metal bracket group and ceramic bracket group. Our finding matches that of the study by Mehmeti et al 19 but contradicts the article by García-Sanz et al 17 This may be due to the design of the metal bracket's base surface, that is, to an uneven surface that creates better mechanical retention compared with ceramic brackets. It would seem that the brackets' mechanical bond to zirconia is stronger than their chemical bond.

Hobson et al 21 defined the lowest acceptable shear strength for routine clinical use as being no less than 5.9 to 7.5 MPa. The values obtained in this study exceed this objective. Ceramill Zolid monolithic zirconia has a bending strength of 700 ± 150 MPa and a Young's modulus ≥ 200 GPa. When debonding the bracket, the risk of causing fractures is therefore small. However, care must be taken with the adhesion between the prosthetic restoration and the tooth, since the strength of this bond depends on several factors, including the type of cementation used. Thus, the right balance must be found to avoid debonding the crown attached to the bracket. The studies have been vague on this issue and have not given any exact maximum strength limit for zirconia crowns. 13

When debonding a bracket, it is important not to alter the structure of the enamel while ensuring that the residual adhesive on the surface of the tooth is minimal. This applies to ceramic materials as well. Our objective during debonding is to minimize cohesive damage to the zirconia and leave as little bonding agent as possible on the surface of the restoration. That is why a low ARI score is desirable, as well as avoiding any cohesive failure within the zirconia. 22

The LMB group displayed the lowest mean ARI score, followed by the SMB, LCB, and SCB groups. The groups with ceramic brackets had higher mean ARI scores than those with metal brackets. However, after analysis, there was no statistically significant difference between these groups.

There are two categories of failure when two indirectly bonded materials come apart:

A cohesive failure occurs within the bonding agent, the bracket, or the zirconia and indicates that the bond in the interface is stronger than that within the material.

An adhesive failure occurs at the bracket/cement or zirconia/cement interface and it indicates that the bond is weaker at the interface between the cement and the material (the zirconia or bracket).

In this study, we found no pure adhesive failures or cohesive failures within the brackets or zirconia blocks, only mixed and cohesive failures within the resin. None of these failures damaged the zirconia or the brackets.

## Conclusion

Within the limits of this study, we can conclude that metal brackets have a greater bond strength than ceramic brackets when cemented to zirconia. No statistically significant difference in shear strength was uncovered between the surface treatments.

As regards ARI score, the sample groups did not appear to have any statistically significant differences between them.

### Authors’ Contributions

Sibel Cetik: Data analysis, manuscript preparation/editing/review.

Thaï Hoang Ha: Literature search, English translation, manuscript review.

Léa Sitri: Experimental studies

Hadrien Duterme: Data acquisition and analysis.

Viet Pham: Date analysis and literature search

Ramin Atash: Concept, design, protocol.

All authors approved the final version of the article.
